# Participation in later life: how civic and political engagement relates to affective well-being

**DOI:** 10.3389/fpubh.2026.1803318

**Published:** 2026-10-13

**Authors:** Joel Bruno da Silva, Leovaldo Alcântara, Teodora Figueiredo, Daniela Melo, Inês Mimoso, Maria Ines Morgado, Joana Carrilho, Luís Midão, Elísio Costa

**Affiliations:** 1 RISE-Health, Competence Centre Active and Healthy Ageing, Faculty of Pharmacy, University of Porto, Porto, Portugal; 2 School of Medicine and Biomedical Sciences, University of Porto, Porto, Portugal; 3 Faculty of Fine Arts, University of Porto, Porto, Portugal

**Keywords:** active ageing, affective well-being, civic and political participation in later life, European Social Survey, older adults

## Abstract

**Inroduction:**

Population ageing has emerged as one of the major demographic challenges of the 21st century, raising important concerns about how mental health can be sustained in later life. In Europe, increasing longevity intersects with social, economic, and institutional inequalities that shape older adults’ opportunities for well-being beyond the absence of disease. Although civic and political participation is widely promoted within active ageing agendas as a positive resource, it is often conceptualised as a homogeneous phenomenon, overlooking the possibility that different forms of engagement may have distinct psychological implications.

**Methods:**

Using data from Round 11 of the European Social Survey (2023), this cross-sectional study examines adults aged 65 years and older across European countries. Affective well-being was operationalised using an index based on the CES-D8. Civic and political participation was captured through four sub-indices (Dutiful, Activating, Expressive and Cooperative profiles) reflecting the typology proposed by Ekman and Amnå. A multiple linear regression model was estimated, controlling for sociodemographic, economic and health-related factors.

**Results:**

The findings indicate that perceived health and household income are strongly associated with affective well-being. Cooperative participation shows the strongest positive association with affective well-being, followed by Expressive and Dutiful participation. In contrast, Activating participation is negatively associated with affective well-being. Cross-national differences were also observed, broadly following a regional pattern, with higher average levels of affective well-being in Northern and Western Europe compared to Southern and Eastern Europe.

**Discussion:**

Taken together, these results suggest that different forms of civic and political participation show distinct patterns of association with affective well-being. Forms of engagement characterised by social embeddedness, reciprocity, and meaningful interaction appear more consistently linked to higher levels of affective well-being, while more confrontational forms of engagement are negatively associated with affective well-being. This differentiated understanding of participation contributes to ongoing debates on active ageing and highlights the importance of context-sensitive strategies to support meaningful civic engagement and mental health among older adults in Europe.

## 1 Introduction

Population ageing has become one of the main challenges of the 21st century. In Europe, it is estimated that by 2050 around 30% of the population will be over 65 years old (1). The European Commission highlights that this trend poses significant challenges for health systems, economic sustainability, social security and, more directly, for the older population, particularly with regard to the promotion of mental health and quality of life (2). In fact, the concept of psychological well-being in ageing has been widely discussed, with different authors proposing different theoretical models to explain it. Among them, the ‘successful ageing’ model proposed by Rowe and Kahn (3) stands out, emphasising the role of health and functional capacity in later life. However, this model has been criticised for its excessive emphasis on individual factors, such as the absence of disability and high cognitive, physical and social performance, thereby excluding individuals with functional limitations, as well as failing to consider the structural and social complexities that shape ageing, being considered an elitist and exclusionary model (4–8). Furthermore, the model is criticised for giving greater visibility to negative mental health indicators, such as depressive symptoms while paying limited attention to positive dimensions of psychological functioning (9, 10).

These limitations have prompted a more holistic reinterpretation of healthy ageing that integrates psychological, social and contextual factors. In this sense, the eudaimonic model of psychological well-being proposed by Carol Ryff (11) emphasises positive psychological functioning, personal fulfilment and meaning in later life. This framework has been widely used in ageing research to understand how psychological and relational resources contribute to healthy ageing beyond the mere absence of disease (11). However, empirical research based on large-scale comparative surveys rarely operationalises psychological well-being through direct measures of all eudaimonic dimensions, often relying instead on specific affective and emotional indicators as proxies for mental health in later life. Accordingly, much of the comparative evidence focuses on emotional well-being and depressive symptomatology as central components of psychological well-being, here interpreted as affective well-being. Despite these advances in research on the subject, there are still gaps on psychological well-being in older adults, especially with regard to multidimensional approaches that integrate emotional, social, and contextual factors (12).

Studies have also suggested that social and political engagement may act as an important psychosocial resource, potentially contributing to a sense of belonging, life purpose and subjective well-being (13–15). However, despite growing recognition of the importance of older adults’ civic and political engagement in agendas and policies, especially in the European context (16), there is a scarcity of studies analysing the influence of civic and political participation on psychological well-being, especially when compared to other topics related to ageing (17–19). The existing studies suggest that active participation and social involvement among older adults are associated with psychological well-being and adaptation at this stage of life (20, 21). Activities such as socialising, physical exercise and community activities have been associated with a reduction in depressive symptoms and an increase in life satisfaction (13, 14, 22, 23, 39).

However, even with advances in studies on the subject, it is clear that participation is understood in a generic and undifferentiated manner, interpreted as a homogeneous phenomenon (24). This type of approach tends to limit understanding of other forms of older adults’ participation in the public sphere, as well as the benefits to psychological well-being associated with them.

The typology proposed by Ekman and Amnå (25) offers a conceptual framework that distinguishes four profiles of civic and political participation: Dutiful, Activating, Expressive, and Cooperative citizens, a synthesis of models that address the transformation of forms of participation, such as those of Dalton (26) and Bennett and Segerberg (27). Different forms of participation may involve distinct psychosocial mechanisms, such as social integration, emotional demands, or exposure to conflict, which may lead to different associations with affective well-being. Specifically, the Dutiful profile corresponds to engagement in conventional and institutional forms, such as institutional trust and confidence in political system. The Activating profile encompasses activities such as participating in protest and confrontation actions, such as demonstrations, signing petitions, and boycotts, aimed at political change. The Cooperative profile is guided by activities focused on the community and collaboration, through volunteering, informal help to neighbours, and participation in local activities. The Expressive profile reflects value-based orientations and normative attitudes, including equality, autonomy, prosocial concern, environmental responsibility, and openness to others. This typology allows us to capture other forms of participation, which may vary according to motivation, intensity, and purpose. However, despite its analytical potential, its application and exploration to understand civic and political participation in the context of European ageing remain limited (28).

Based on this framework, the present study aims to analyse, in a European context, how different profiles of civic and political participation, as proposed by Ekman and Amnå (25) are associated with the affective dimension of psychological well-being of older adults. Using data from the European Social Survey (ESS), this study seeks not only to extend the empirical application of this typology to later life, but also to explore how distinct forms of participation are differently associated with psychological well-being, while controlling for key sociodemographic and health-related factors.

## 2 Methods

### 2.1 Study design and data

This study adopts a quantitative, cross-sectional, and exploratory approach. The data used are from the 11th round (2023) of the ESS, a biannual survey that measures the attitudes, beliefs, and social behaviours of the population residing in European countries (29). The ESS provides detailed information on sociodemographic variables, health, socioeconomic status, and various forms of social, civic, and political participation, making it a suitable data source for the research objectives.

The 11th round includes data from participating European countries as well as Israel. For the purposes of this study, the analysis was restricted to European countries, excluding Israel. The data were collected through computer-assisted personal interviews (CAPI), following methodological protocols that ensure cross-national comparability. These protocols include probabilistic sampling designs, strict fieldwork procedures, interviewer training, translation and back-translation of questionnaires, and the use of post-stratification and design weights to correct sampling and non-response biases (55).

To ensure cross-national comparability and account for sampling design and population structure, combined ESS weights (design and post-stratification weights) were applied in all analyses. The combined ESS weight was computed by multiplying the design weight (dweight) by the post-stratification weight (pspwght) and subsequently normalised to have a mean of 1. Due to listwise deletion in the regression models, the final analytical sample used in multivariate analyses was 12,348 cases.

### 2.2 Participants

The original sample for the 11th Round of the ESS consisted of 46,162 individuals. The following sequential inclusion criteria were applied:

1. Residence in a European country.
2. Age 65 years or older.

After applying these criteria, the sample consisted of 13,020 participants.

In a second step, cases with missing or invalid responses in the variables required to construct the affective well-being index and participation sub-indices were excluded. As a result, the number of valid cases varied across analyses due to different patterns of missing data across variables. For example, the number of valid cases for the affective well-being index (CES-D8-based measure) was 12,997, while the participation sub-indices ranged between 12,827 and 13,016, depending on the specific items included (see Figure 1 for details). For the multivariate analyses, listwise deletion was applied, resulting in a final analytical sample of 12,348 cases with complete data across variables included in the regression model. Participants were then categorised into three age groups for comparative analyses: 65–74 years, 75–84 years, and 85 years or older. Although ESS round 11 includes a larger number of participating countries, the country-level analyses in this study were restricted to countries with valid observations for respondents aged 65 years and older and complete data on the affective well-being index. Consequently, the number of countries included in the country-level analyses is lower than the total number of European countries participating in the survey, due to data availability constraints.

**Figure 1:**
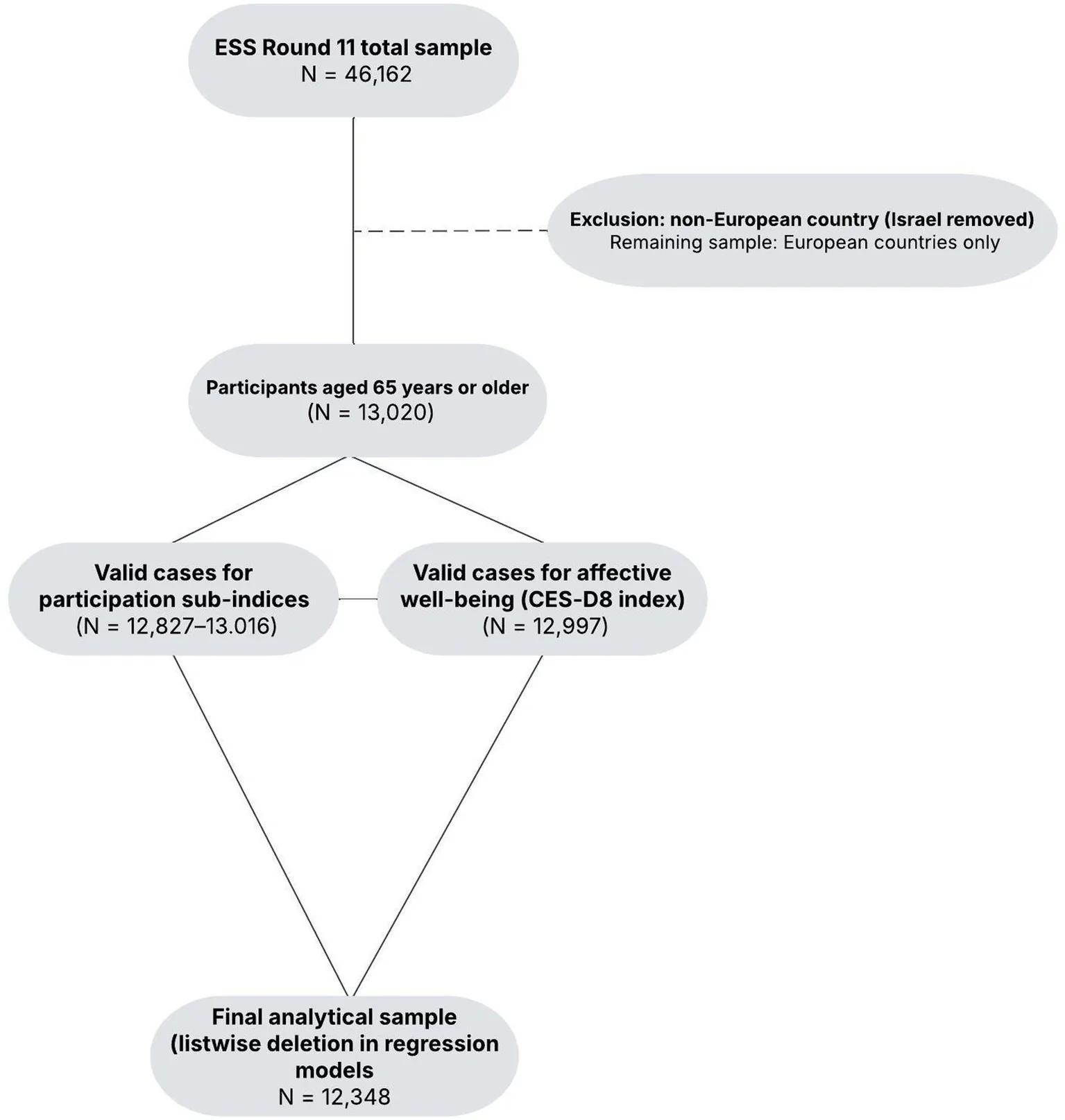
Flowchart of sample selection process and derivation of the final analytical sample based on ESS Round 11 data.

### 2.3 Measures and variables

#### 2.3.1 Dependent variable: affective well-being

In line with large-scale comparative ageing research, which often prioritises the affective dimension of well-being due to its relevance in later life (30), affective well-being was operationalised using the eight items of the Centre for Epidemiologic Studies Depression Scale (CES-D8), developed by Radloff (31). All items refer to the frequency of emotional states experienced during the week prior to the interview and are measured on a four-point scale ranging from 1 (never or rarely) to 4 (always or almost always).

Six CES-D8 items capturing negative affect were reverse-coded (felt depressed, felt that everything you did was an effort, felt sad, felt lonely, sleep was restless, could not get going) so that higher values indicate fewer depressive symptoms, while the two positive affect items (felt happy, enjoyed life) were already positively oriented. A continuous affective well-being index was computed as the mean of the eight items using the SPSS MEAN.4 function, requiring a minimum of four valid responses. The resulting scale ranges from 1 to 4, with higher values indicating higher levels of affective well-being, and showed good internal consistency in the present sample (Cronbach’s α = 0.77).

While this operationalisation does not capture the full range of eudaimonic dimensions of well-being, consistent with previous cross-national research, the CES-D8 has been shown to be strongly and inversely associated with positive well-being constructs (32–34), supporting its use as a reliable proxy for affective well-being in comparative studies.

#### 2.3.2 Main independent variables: civic and political participation profiles

Social, civic, and political participation was operationalised based on the typology proposed by Ekman and Amnå (25), which distinguishes four profiles of engagement: Dutiful, Activating, Expressive and Cooperative. Based on this framework, four continuous sub-indices were constructed using variables available in the ESS that best capture the defining behaviours and orientations of each profile. The selection of items for each sub-index was guided by both theoretical alignment and data availability within the ESS.

For each sub-index, items were recoded so that higher values consistently represented higher levels of participation or predisposition to engage. Composite indices were calculated as the mean of the variables that comprise each dimension applying a minimum valid response criterion (at least 50% of items per index). The resulting indices were subsequently normalised to a scale ranging from 0 to 1 to ensure comparability across dimensions. The Dutiful profile captures attitudinal engagement through institutional trust, including trust in political institutions, such as parliament, the legal system, politicians, and political parties. The Activating profile captures non-institutional and action-oriented forms of political participation, such as contacting a political representative, participating in political parties or pressure groups, signing a petition, boycotting products, and engaging in political actions both offline and online. The Expressive profile represents attitudinal and symbolic engagement with politics, including value-based orientations related to equality, autonomy, prosocial concern, environmental responsibility, and openness to others. This index reflects engagement through value-based orientations and normative positioning, rather than direct action. The Cooperative profile captures forms of social participation and community engagement, including frequency of social interaction, participation in social activities, and availability of close personal support networks. This index reflects relational embeddedness and everyday forms of social involvement. To ensure comparability across participation profile, sub-indices were normalised to a 0–1 scale. Specifically, indices with different original ranges were rescaled using min-max transformation, so that higher values consistently represent higher levels of participation.

Table 1 summarises the composition and logic of each sub-index, while the full list of constituent variables is provided in the Supplementary material. Internal consistency analyses indicated acceptable reliability for all sub-indices, supporting their use as composite measures of distinct participation profiles.

**Table 1:** Operationalisation of civic and political participation profiles.

Sub-index (Profile)	Central concept	Examples of behaviours/Attitudes measured	Construction procedure
*Dutiful*	Civic duty and institutional engagement	Trust in political institutions (e.g., parliament, legal system, politicians, political parties).	Mean of recoded items (minimum 50% valid responses), normalised to a 0–1 scale.
*Activating*	Active and confrontational mobilisation	Signing petitions, participating in demonstrations, boycotting products, contacting a political representative, participating in political parties or pressure groups, and posting and sharing political content online (e.g., social media).	Mean of recoded items (minimum 50% valid responses), normalised to a 0–1 scale.
*Expressive*	Value-based orientations and normative engagement	Value-based orientations related to social responsibility and prosocial attitudes (e.g., equality, autonomy, caring for others, environmental concern, openness to others).	Mean of recoded items (minimum 50% valid responses), normalised to a 0–1 scale.
*Cooperative*	Collaborative community involvement	Frequency of social interaction, participation in social activities, and availability of close personal support networks.	Mean of recoded items (minimum 50% valid responses), normalised to a 0–1 scale.

Full list of variables used in each sub-index is provided in Supplementary Table S1.

Correlations between the sub-indices were low to moderate, indicating that although related they capture distinct dimensions, in line with the underlying theoretical framework.

#### 2.3.3 Control variables

To isolate the effect of participation profiles and obtain more robust estimates, a set of sociodemographic, economic, health, and contextual variables was included in the regression analyses. These comprised sociodemographic characteristics (age group 65–74, 75–84, and 85+; gender; marital status; level of education, based on ISCED recoded into low, medium, and high levels); economic conditions (perceived household income); health status (self-reported subjective health and limitations in daily activities due illness, disability, or infirmity). In addition, contextual variables included household size (number of people living regularly in the household). All control variables were recoded and categorised as appropriate for statistical analyses.

#### 2.3.4 Data analysis strategy

All analyses were conducted using IBM SPSS Statistics software (version 29.0). To ensure cross-national comparability and account for sampling design, analyses were performed using combined ESS weights (design and post-stratification weights), normalised to a mean of 1. Descriptive statistics (frequencies, means, and standard deviations) were used to characterise the sample. Differences in affective well-being index and participation sub-indices were explored using one-way ANOVA, with Tukey HSD or Games–Howell post-hoc tests applied depending on the assumption of homogeneity of variances (Levene’s test). Effect sizes were reported using partial η^2^. Bivariate associations between participation profiles and affective well-being were initially examined using Pearson correlation coefficients.

To assess the association between civic and political participation and affective well-being, a multiple linear regression model was estimated with affective well-being as the dependent variable. The model included sociodemographic variables (age group, gender, education level, marital status), economic conditions (perceived household income), health-related variables (subjective health and limitations in daily activities), and household characteristics (household size), alongside the four participation sub-indices (Dutiful, Activating, Expressive, and Cooperative). This modelling strategy allows estimation of the independent contribution of each participation profile, while controlling for structural, economic, and health-related factors. Model assumptions, including normality of residuals, homoscedasticity and multicollinearity, were assessed. Variance inflation factors (VIF) were examined to detect potential multicollinearity issues. Statistical significance was set at *p* < 0.05.

## 3 Results

### 3.1 Characterisation of the sample

After selecting respondents aged 65 years or older, the analytical sample consisted of 13,020 participants. Due to missing data across variables, the effective sample size varied between analyses, with 12,348 valid cases available when considering sociodemographic, economic, health, and contextual variables jointly. For the regression models, listwise deletion was applied, resulting in a final analytical sample of 12,348 cases. The sample included respondents from 25 European countries participating in ESS Round 11. Country-level analyses were restricted to countries with sufficient valid cases for respondents aged 65 years or older and complete data on the key study variables. These countries covered Northern, Southern, Eastern, and Western European regions.

Table 2 summarises the main sociodemographic, health-related, and contextual characteristics of the sample. The gender distribution was relatively balanced, with 54.7% women and 45.3% men (M = 0.53; SD = 0.50). Participants were distributed across three age groups, with 57.4% aged 65–74, 34.2% aged 75–84, and 8.5% aged 85 or older (M = 74.09; SD = 6.57).

**Table 2:** Sociodemographic characteristics and health characteristics of the sample.

Category	*N*	%
Gender		
Female	7,128	54.7
Male	5,892	45.3
Age group		
65–74	7,469	57.4
75–84	4,449	34.2
85+	1,102	8.5
Level of education		
Low education	4,691	36.3
Medium education	4,576	35.4
High education	3,672	28.4
Marital status		
Married/Cohabiting	7,228	56.8
Widowed	3,441	27
Divorced/Separated	1,304	10.2
Never married	763	6.0
Number of people in the household		
Lives alone	4,853	37.4
Two- person household	6,914	53.3
With 2 + persons	1,204	9.3
Perceived income		
Comfortable	4,005	31.0
Coping financially	5,728	44.3
Difficult	2,380	18.4
Very difficult	803	6.2
Perceived health		
Good health	5,863	45.1
Fair health	5,236	40.3
Poor health	1,902	14.6

Values correspond to valid cases for each variable. Differences in sample size (*n*) reflect missing data across variables. The analytical sample includes respondents aged 65 years and older. For multivariate analyses, listwise deletion was applied, resulting in a final analytical sample of 12,348 cases.

Regarding educational attainment (ISCED), the sample showed a relatively balanced distribution (M = 1.85; SD = 0.80). Most respondents were married or in a civil partnership, followed by widowed, divorced/separated, and never married individuals (M = 2.49; SD = 0.89). In terms of health conditions, participants reported moderate levels of subjective health (M = 1.70; SD = 0.71), while limitations in daily activities showed moderate distribution (M = 0.54; SD = 0.69). Regarding economic conditions, the perceived household financial situation indicated an intermediate position (M = 1.99; SD = 0.84). In terms of household composition, the number of people living in the household showed moderate variation (M = 1.91; SD = 0.65).

Affective well-being was assessed using a composite index ranging from 1 to 4, with higher values indicating higher levels of affective well-being. Overall, affective well-being in the sample was relatively high (M = 3.21; SD = 0.55), indicating relatively high levels of affective well-being.

Table 3 presents the descriptive statistics for the four participation sub-indices (all sub-indices range from 0 to 1), noting that the number of valid cases varies across measures due to item-specific missingness. The Expressive profile sub-index showed the highest mean values (M = 0.80; SD = 0.12), indicating strong endorsement of value-based orientations related to social responsibility, equality, and prosocial concern. The Dutiful profile sub-index also showed moderate values (M = 0.42; SD = 0.23), reflecting institutional trust and attitudinal engagement with political institutions. Cooperative participation showed intermediate levels (M = 0.46; SD = 0.19), reflecting social connectedness and relational embeddedness. In contrast, the Activating profile sub-index showed the lowest values (M = 0.10; SD = 0.18), indicating relatively limited engagement in protest-oriented or confrontational forms of participation.

**Table 3:** Descriptive statistics of participation measures.

Sub-index	*N*	M	SD
Expressive profile	12,827	0.80	0.12
Dutiful profile	12,917	0.42	0.23
Cooperative profile	12,993	0.46	0.19
Activating profile	13,016	0.10	0.18

All sub-indices were normalised to a 0–1 scale, with higher values indicating levels of participation. Differences in sample size (N) reflect item-specific missing data.

Cross-national variation in affective well-being was observed, as shown in Table 4, which summarises the distribution of affective well-being across European countries. The results indicate that mean values differ between countries, although most national averages fall within a relatively narrow range around the overall mean (M = 3.21; SD = 0.55). These descriptive patterns highlight the heterogeneity of affective well-being among older adults in Europe and provide a basis for subsequent comparative analyses (Table 5).

**Table 4:** Distribution of affective well-being across European countries.

Country	*N*	Mean	SD
Austria	634	3.39	0.43
Belgium	442	3.33	0.50
Bulgaria	715	2.96	0.64
Croatia	609	3.11	0.56
Cyprus	204	3.21	0.55
Finland	535	3.40	0.44
France	485	3.34	0.54
Germany	715	3.28	0.49
Greece	807	2.93	0.47
Hungary	630	2.96	0.55
Ireland	363	3.51	0.43
Iceland	84	3.49	0.46
Italy	1,173	3.06	0.51
Latvia	494	2.99	0.50
Lithuania	340	3.04	0.53
Netherlands	477	3.44	0.47
Norway	347	3.50	0.41
Poland	475	3.31	0.55
Portugal	389	3.08	0.61
Serbia	535	3.16	0.56
Slovakia	333	3.06	0.48
Slovenia	359	3.29	0.52
Sweden	371	3.42	0.44
Total	12,997	3.21	0.55

The table includes only countries with valid observations for respondents aged 65 years and older and complete data on affective well-being. Differences in samples size (N) reflect item-specific missing data.

**Table 5:** Descriptive statistics of affective well-being by European region.

Region	*N*	Mean	SD	95% CI
North	1,337	3.44	0.43	[3.42–3.45]
South	3,067	3.07	0.55	[3.05–3.09]
West	3,962	3.38	0.48	[3.37–3.39]
East	4,632	3.08	0.57	[3.06–3.10]
Total	12,997	3.21	0.55	[3.20–3.21]

Values correspond to regional means of affective well-being. Differences in N reflect item-specific missing data across regions.

Higher values of affective well-being were observed in Northern (M = 3.44, 95% CI [3.42–3.45]) and Western Europe (M = 3.38, 95% CI [3.37–3.39]), while lower mean values were found in Southern (M = 3.07, 95% CI [3.05–3.09]) and Eastern European regions (M = 3.08, 95% CI [3.06–3.10]). These patterns indicate the presence of regional differences in affective well-being among older adults in Europe, with a consistent gradient favouring Northern and Western regions.

### 3.2 Multiple linear regression analysis

A multiple linear regression analysis was conducted to examine the association between sociodemographic, economic, health-related and participation variables and affective well-being. The model explained a moderate-to-substantial proportion of the variance in affective well-being (R^2^ = 0.398; *p* < 0.001). Among the predictors, subjective health showed the strongest association with affective well-being (β = −0.243; *p* < 0.001), followed by perceived household income (β = −0.211; *p* < 0.001) and limitations in daily activities (β = −0.155; *p* < 0.001), suggesting that better health and financial conditions are associated with higher levels of affective well-being.

Regarding sociodemographic variables, women reported slightly lower levels of affective well-being than men (β = −0.090; *p* < 0.001), while age showed a small negative association (β = −0.020; *p* = 0.009). Education level was not significantly associated with the outcome (β = 0.005; *p* = 0.519). Household size showed a small but significant positive association (β = 0.033; *p* < 0.001). Regarding civic and political participation, Cooperative participation showed the strongest positive association with affective well-being (β = 0.192; *p* < 0.001), followed by Expressive (β = 0.091; *p* < 0.001) and Dutiful participation (β = 0.035; *p* < 0.001). In contrast, Activating participation was negatively associated with affective well-being (β = −0.044; *p* < 0.001).

Variance Inflation Factors (VIF) were examined and indicated no issues of multicollinearity (all VIF values < 2) (Table 6).

**Table 6:** Multiple linear regression predicting affective well-being.

Variables	*β*
Age (65+)	−0.020*
Gender (female)	−0.090***
Education (ISCED)	0.005
Marital status	−0.094***
Household income	−0.211***
Self-rated health	−0.243***
ADL limitations	−0.155***
Number of people in household	0.033***
Dutiful profile	0.035***
Activating profile	−0.044***
Expressive profile	0.091***
Cooperative profile	0.192***
R^2^	0.398
*N*	12,348

β, standardised coefficients. *p < 0.05, **p < 0.01, ***p < 0.001.

## 4 Discussion

This study aimed to investigate the relationship between different profiles of civic and political participation and affective well-being among adults aged 65 and above in Europe, while controlling for key structural factors. The results reveal a consistent pattern: structural conditions, particularly perceived health and financial situation, are strongly associated with affective well-being in later life. However, even after accounting for these influences, different types of participation show statistically significant associations of varying magnitude of affective well-being, highlighting important differences across forms of engagement. Beyond confirming the importance of structural determinants, this study contributes to the literature by showing that civic and political participation in later life is not a homogeneous phenomenon; rather, its associations with affective well-being appear to vary according to the type of citizenship enacted. By situating the findings within the eudaimonic framework (11) and the typology proposed by Ekman and Amnå (25), this study offers a more differentiated interpretation of how active ageing relates to affective well-being, while acknowledging that the outcome primarily reflects the affective dimension of well-being.

### 4.1 The primacy of structural conditions: health and resources

As expected, and in line with the literature (35, 36), perceived health and household income emerge as the factors most strongly associated with affective well-being in later life. These findings reinforce that any discussion of active ageing and civic participation must acknowledge the central role of structural conditions in shaping quality of life in older age. Good health constitutes a fundamental prerequisite for positive psychological functioning, as it enables autonomy, mobility, and engagement in socially meaningful activities. These capacities are closely related to dimensions such as environmental mastery and autonomy in the framework proposed by Ryff (11). Similarly, economic security provides the structural foundation that reduces material vulnerability and expands opportunities for social and civic engagement. These findings challenge overly individualistic perspectives on ‘successful ageing’, such as those proposed by Rowe and Kahn (3), emphasising instead that socioeconomic and health conditions remain strongly associated with well-being in later life (as captured by affective well-being).

It is also important to recognise that cognitive functioning and functional capacity may contribute to maintaining the affective well-being of older adults at this stage of life, even after accounting for depressive symptoms (37). Overall, these findings suggest that affective well-being in ageing is strongly embedded in structural and functional conditions accumulated across the life course. In this context, civic and political participation should be understood as a complementary factor that may be associated with higher levels of affective well-being, rather than a substitute for health and socioeconomic resources.

### 4.2 Differentiated participation: the role of civic duty and cooperation

Among the different profiles of civic and political participation analysed, Dutiful and Cooperative profiles showed a positive association with affective well-being. The Dutiful profile, characterised by conventional and duty-based forms of engagement such as institutional trust, reflecting confidence in political institutions such as parliament, political parties, and the legal system, was positively associated with affective well-being (β = 0.035). This pattern suggests that such forms of participation may reflect broader processes of social integration and normative stability. For many older adults, these forms of engagement may reinforce a sense of belonging to the social system and continuity of civic identity across the life course. The mechanisms are consistent with eudaimonic perspectives that emphasise purpose in life and social embeddedness as relevant dimensions of well-being (11, 38). Similarly, Cooperative participation, operationalised through indicators of social interaction, participation in social activities, and availability of close personal networks, was positively related to affective well-being (β = 0.192). The relatively stronger association observed for Cooperative participation reflects the socially embedded and interaction-oriented nature dimension. This form of engagement may operate through several psychosocial mechanisms, including the strengthening of social networks, the experience of reciprocity, and a sense of social belonging and interpersonal connectedness. These mechanisms are closely related to the development of social capital and supportive interpersonal relationships, which have consistently been identified as protective factors for affective well-being in later life (39, 40). Participation in socially embedded activities may also reduce feelings of isolation while reinforcing perceptions of usefulness, recognition, and social integration.

In addition, forms of participation grounded in cooperation and prosocial engagement may contribute to a stronger sense of meaning and purpose in life, dimensions that are central to eudaimonic perspectives of well-being (11). Recent research suggests that civic participation may reinforce growth-oriented and prosocial values associated with belongingness, community involvement, and social contribution, which in turn may positively relate to well-being in later life (41). Although these associations vary in magnitude, they point to specific pathways through which participation may relate to affective well-being, particularly when it involves meaningful social interaction and recognition. More broadly, the literature suggests that potential benefits of social engagement depend on its type, intensity, and subjective meaning (42, 43), reinforcing the need to move beyond undifferentiated notions of participation.

### 4.3 More complex or differentiated forms of participation

The remaining participation profiles reveal more complex patterns. The Activating profile showed a small but statistically significant negative association with affective well-being (β = −0.044), suggesting that more contentious or mobilisation-oriented forms of participation may be linked to lower levels of affective well-being in later life. This profile encompasses a broad range of action-oriented political behaviours, including both institutional engagement (e.g., contacting political representatives and participating in political organisations) and non-institutional forms (e.g., protests, boycotts, and online political expression). Although this finding may initially appear counterintuitive, it may reflect that engagement in protest-oriented or high-intensity political activities can involve emotional demands, exposure to conflict, political frustration, or perceived lack of efficacy. Recent literature has increasingly described these dynamics in terms of the ‘emotional cost of activism’, particularly in contexts characterised by political polarisation, sustained sociopolitical (44), particularly in contexts of sustained political tension, or intense exposure to political conflict (45). In such contexts, participation in contentious or confrontational forms of engagement may involve chronic emotional demands, frustration, and heightened vigilance, and feelings of inefficacy, all of which may contribute to emotional exhaustion and psychological strain over time.

Previous research has also shown that certain forms of political participation and political group involvement may be associated with higher levels of depressive symptoms among older adults, especially when participation occurs in highly polarised or conflict-oriented environments (22, 46). These effects may partly reflect the cognitive and emotional burden associated with sustained engagement in contentious political contexts, particularly when individuals perceive low institutional responsiveness or limited social reciprocity. In this sense, while activism may represent an important form of civic expression and democratic engagement, it may simultaneously generate psychosocial costs that negatively affect affective well-being in later life. In addition, sociopolitical unrest and prolonged exposure to contentious political environments may negatively affect older adults´ mental well-being, contributing to emotional distress, insecurity, and disruption of social relationships (47). In this sense, participation in politically contentious environments may not necessarily operate as a protective psychosocial resource, particularly when engagement occurs in contexts marked by instability, conflict, or low perceived responsiveness of institutions. At the same time, the cross-sectional nature of the data does not allow causal interpretation. It is possible that individuals experiencing lower levels of affective well-being or higher dissatisfaction are more likely to engage in such forms of participation, reflecting possible reciprocal or bidirectional dynamics between political mobilisation and affective well-being rather than a simple unidirectional association (48).

Expressive participation was positively associated with affective well-being (β = 0.091), although with a modest effect size. This profile reflects value-based orientations, including equality, autonomy, prosocial concern, and openness to others, rather than direct forms of political behaviour.

These findings suggest that holding prosocial and socially oriented values may be positively associated with affective well-being, although to a lesser extent than forms of participation involving direct social interaction. This interpretation aligns with previous research indicating that not all forms of participation yield equivalent outcomes and that some may be more weakly or context-dependently associated in their relationship with affective well-being (24).

Taken together, these results reinforce the idea that in later life, the relationship between participation and affective well-being is differentiated, and that forms of engagement involving social embeddedness and mutual exchange appear more consistently associated with higher levels of affective well-being.

### 4.4 The regional gradient: context as a key determinant

The results revealed a regional pattern, with comparatively higher levels of affective well-being observed in Northern and Western Europe, and lower levels in Southern and Eastern Europe. These differences should not be interpreted as intrinsic regional characteristics, but rather as the outcome of long-standing institutional, welfare and socio-political configurations. More specifically, these regional differences may partly reflect variations in welfare state regimes and in the degree of de-familiarisation across European contexts. Previous comparative research has identified a consistent north–south gradient in productive ageing activities across Europe, suggesting that welfare regimes characterised by higher levels of de-familiarisation and stronger social protection systems tend to create more favourable conditions for participation in later life (49). Nordic welfare regimes, characterised by stronger public welfare provision, higher levels of social protection, and more developed civic infrastructure, may reduce structural barriers to participation by expanding opportunities for social engagement beyond the family sphere. In contrast, Mediterranean welfare regimes have historically relied more heavily on family-based support systems and informal solidarity networks, often with comparatively lower levels of public investment in civic and community infrastructure.

This pattern highlights the importance of contextual factors beyond individual characteristics. It reflects differences in the robustness of welfare states, levels of social capital, and institutional trust, which are generally more consolidated in Northern European countries. In these contexts, historically sustained investments in social protection and civic infrastructure may help explain higher levels of participation and affective well-being, supported by environments of high social trust (50, 51). The presence of institutional arrangements that function as participation infrastructure, such as associations, public support services, and community spaces, appears to play a central role in enabling civic and political engagement over time. As Martins (52) argues, these socio-political trajectories have lasting effects not only on levels of participation in later life, but also on how individuals experience and engage with civic roles. From this perspective, active ageing should be understood not merely as an individual disposition, but as a socially produced opportunity structure shaped by welfare institutions, labour market trajectories, and civic infrastructure accumulated over the life course.

In this sense, the findings suggest that the promotion of well-being and participation in later life transcends a strictly individualistic perspective, demanding the strengthening of social, economic, and political institutional ecosystems capable of supporting structured and meaningful opportunities for engagement throughout the ageing process. This understanding aligns with an age-friendly ecosystems approach, which prioritises the creation of supportive social and community environments that facilitate participation and inclusion in later life (53). Such a framework reflects an ecological understanding of active ageing that recognises the primacy of contextual conditions over individual factors, as advocated by the World Health Organization within the Decade of Healthy Ageing (54) and the European Commission’s Green Paper on Ageing (2).

### 4.5 Limitations

This study has several limitations that should be considered when interpreting the findings. First, its cross-sectional design prevents causal inference, making it impossible to establish the direction of the associations observed between civic and political participation and affective well-being. It is therefore plausible that higher levels of affective well-being may facilitate civic and political engagement, just as participation itself may contribute to affective well-being, suggesting the possibility of bidirectional or reciprocal relationships. In this sense, affective well-being may function not only as an outcome of participation, but also as a psychosocial resource that facilitates continued social engagement over time, particularly in more socially embedded forms of participation. This is particularly relevant given that different forms of participation showed distinct associations with affective well-being.

Second, the outcome variable was operationalised as an index based on the CES-D8, which primarily captures the affective dimension of well-being and emotional distress. Although this approach is widely used in comparative ageing research, it does not fully capture the broader range of eudaimonic dimensions, such as purpose in life, personal growth, autonomy, or self-realisation, as conceptualised in Ryff’s (11) framework. Therefore, the findings should be interpreted as reflecting associations with affective well-being rather than psychological well-being in its broader sense. In addition, the absence of depressive symptoms should not necessarily be interpreted as the presence of flourishing or optimal psychological functioning. Some psychosocial effects associated with civic and political participation, particularly those related to meaning, identity, social contribution, and purpose in life, may not be fully captured by the outcome measure adopted in this study.

Third, the data are based on self-reported measures, which may be subject to recall bias and social desirability effects. In addition, the ESS sample may underrepresent older adults in more vulnerable situations, including those with severe health limitations or living in institutional settings, which may limit the generalisability of the findings.

Fourth, although the participation sub-indices provide a useful analytical framework for distinguishing different profiles of engagement, they do not fully capture the intensity, subjective meaning, voluntariness, or lived experience of participation, all of which may shape how civic and political engagement relates to affective well-being in later life. Furthermore, the construction of composite indices may obscure variation within each participation profile.

Finally, the use of pooled cross-national data introduces additional analytical complexity, as differences between countries in institutional contexts, cultural norms, and welfare systems are not fully captured by the analytical approach adopted. Although regional patterns were explored descriptively, further research using more advanced strategies, such as multilevel modelling and longitudinal designs, would be necessary to better account for country-level variation and clarify the temporal dynamics between participation and affective well-being.

### 4.6 Directions for future research

The findings and limitations of this study point to several promising avenues for future research. First, longitudinal or panel designs are needed to disentangle the potentially bidirectional relationship between participation profiles and affective well-being, allowing for a more robust understanding of how participation and affective well-being may influence each other over time.

Second, it would be valuable to replicate this analysis using direct measures of positive well-being, such as the scales proposed by Ryff (11), to complement the affective perspective captured by the CES-D8 and assess the robustness of the associations observed across different conceptualisations of well-being. Third, further research should explore in greater depth the mechanisms through which different forms of participation relate to affective well-being. In particular, qualitative and mixed-methods approaches could provide insight into psychosocial processes such as social support, self-efficacy, emotional regulation, and perceived social recognition. This would be especially relevant for understanding the more complex patterns observed in the Activating and Expressive profiles, including the potential emotional demands associated with confrontational forms of participation and the relatively modest effects observed for more discursive forms of engagement.

Finally, future research should further explore cross-national analyses, examining how specific configurations of the welfare regimes, institutional contexts, and political cultures shape the relationship between participation and affective well-being. Approaches that explicitly account for country-level variation, such as multilevel modelling, would be particularly valuable to understanding how macro-level conditions moderate individual-level associations. Such efforts would contribute to the development of more context-sensitive and effective strategies for promoting active and healthy ageing across Europe.

### 4.7 Implications for policy and practice

The findings of this study suggest that policies promoting active and healthy ageing should not rely solely on individual motivation or behavioural change. Instead, the strong associations observed for perceived health, financial security, and regional context indicate that engagement in civic and community activities in later life is closely associated with structural conditions. By moving beyond a generic understanding of participation, this study highlights that different forms of engagement show distinct patterns of association with affective well-being. In particular, forms of participation that involve social interaction, reciprocity, and a sense of contribution, such as Cooperative and, to a lesser extent, Dutiful forms of engagement, appear more consistently linked to higher levels of affective well-being. These findings suggest that policy interventions should prioritise creating opportunities for meaningful and socially embedded participation, rather than simply increasing participation rates overall.

First, integrated approaches are required. Policies should combine support for health and economic security with the development of structured opportunities for civic and community engagement. Without addressing these underlying conditions, participation initiatives risk primarily benefiting those who are already healthier and more socioeconomically advantaged.

Second, policy design should explicitly account for regional disparities. The lower levels of affective well-being observed in Southern and Eastern Europe point to the need for broader investments in social cohesion, institutional trust, and access to community-based infrastructures. In these contexts, strengthening local civic ecosystems and reducing structural inequalities may be a necessary precondition for increasing meaningful participation.

Third, participation opportunities should be differentiated according to their form and potential impact. Local authorities and civil society organisations should prioritise initiatives that foster social embeddedness and mutual exchange, such as volunteering programmes, intergenerational initiatives, and community-based activities. At the same time, it is important to recognise that different forms of engagement may have distinct implications for well-being, and that not all types of participation generate equivalent psychosocial benefits.

Taken together, these implications suggest that promoting well-being in later life requires a shift from individual-centred approaches to more systemic strategies that strengthen the social and institutional conditions under which participation occurs.

### 4.8 Policy recommendations

Based on these findings, particularly the differentiated patterns observed across participation profiles and regional contexts, the following practical recommendations are proposed for policymakers, local authorities, and civil society organisations.

1. Prioritise forms of participation associated with social embeddedness and reciprocity. Expand opportunities for socially embedded and community-oriented forms of engagement, such as structured volunteering, neighbourhood associations, and intergenerational programmes. Co-design participation initiatives with older adults to ensure they promote meaningful involvement, recognition, and a sense of contribution.
2. Strengthen inclusive participation infrastructures. Invest in accessible community centres, civic hubs, and local networks that function as entry points for participation. Examples may include intergenerational community centres, neighbourhood participation networks, local volunteering hubs, and age-friendly civic spaces designed to facilitate social interaction and community engagement among older adults. Ensure that these environments are physically, economically, and cognitively accessible.
3. Promote coordinated local governance partnerships. Encourage collaboration between municipalities, community organisations, and civil society actors to develop context-sensitive participation initiatives aligned with local needs and participation profiles. This may include municipal senior councils, participatory governance initiatives involving older adults, and locally coordinated volunteering programmes that actively integrate older residents into community decision-making processes.
4. Address regional inequalities through context-sensitive strategies. In Southern and Eastern European contexts, prioritise policies that strengthen welfare provision, reduce economic insecurity, and rebuild institutional trust, as these factors appear to be associated with both participation and well-being. In contrast, in Northern and Western contexts, efforts may focus more on sustaining and diversifying existing participation infrastructures.
5. Support lifelong civic and digital inclusion. Develop programmes that enhance civic literacy, digital skills, and access to information, enabling older adults to engage more effectively in both community-based and mediated forms of participation. Particular attention should be given to ensuring that digital and expressive forms of participation are accessible and meaningful, while recognising that different forms of engagement may have distinct implications for well-being, particularly given the stronger associations observed for more socially embedded forms of participation.

Taken together, these recommendations reinforce the importance of developing sustainable participation infrastructures capable of supporting meaningful engagement opportunities throughout the ageing process.

## 5 Conclusion and implications

This study shows that the relationship between civic and political participation and affective well-being in later life is differentiated and highly contextual. Not all forms of participation are associated with affective well-being in the same way, and their potential implications appear to depend on the type of engagement involved, as well as on the broader social and institutional conditions in which participation occurs. The findings suggest that, while health and economic resources constitute the fundamental basis for affective well-being in later life, certain forms of participation, particularly those characterised by social embeddedness, reciprocity, and meaningful interpersonal interaction, are more consistently associated with higher levels of affective well-being. In this regard, Cooperative participation showed the strongest positive association, followed by Expressive and Dutiful forms of engagement. These forms of participation may reflect processes of social integration, belongingness, purpose, and social recognition that contribute to emotional well-being in ageing.

In contrast, more contentious or mobilisation-oriented forms of participation were negatively associated with affective well-being, suggesting that participation may also involve emotional demands, political frustration, or psychosocial strain, particularly in contexts characterised by conflict, instability, or low institutional responsiveness. Taken together, these findings reinforce the importance of moving beyond undifferentiated understandings of civic participation in later life. Rather than treating participation as a uniformly beneficial process, the results support a more ecological and context-sensitive perspective, recognising that the relationship between participation and affective well-being depends not only on whether individuals participate, but also on how they participate and within which social, institutional, and welfare contexts participation is embedded.

## Data Availability

Publicly available data were analysed in this study. Data from the European Social Survey (ESS) Round 11 – 2023 were used and can be accessed through the ESS Data Portal (56).
