# Supplementary material for: Participation in later life: how civic and political engagement relates to affective well-being
Source: Front Public Health. 2026 Oct 13;14:1803318. doi: 10.3389/fpubh.2026.1803318 (PMC13710365; doi:10.3389/fpubh.2026.1803318)
Supplement: Supplementary file 1 [file Data_Sheet_1.docx]

**Supplementary Material**

**Table S1**. Full list of ESS variables included in each sub-index.

| Profile | Variables used |
| --- | --- |
| Dutiful | -Trust in the European Parliament  -Trust in the legal system  -Trust in politicians  -Trust in Political Parties |
| Activating | -Boycotted certain products in the last 12 months  -Contacted a politician or government official in the last 12 months  -Donated to or participated in a political party or pressure group in the last 12 months  -Signed a petition in the last 12 months  -Taken part in a public demonstration in the last 12 months  -Posted or shared anything about politics online in the last 12 months |
| Expressive | -Important that people are treated equally and have equal opportunities  -Important to make own decisions and be free  -Important to help people and care for others’ well-being Important to care for nature and environment  -Important to understand different people |
| Cooperative | -How often socially meet with friends  -Take part in social activities compared to others of same age  -How many people with whom you can discuss intimate and personal matters |

**Table S2**. Internal consistency of participation sub-indices

| Sub-Index | Cronbach’s alpha | Number of items |
| --- | --- | --- |
| Dutiful profile | 0.889 | 4 |
| Activating profile | 0.626 | 6 |
| Expressive profile | 0.689 | 5 |

| Cooperative profile | 0.564 | 3 |
| --- | --- | --- |

**Note:** Lower alpha values for the Cooperative profile may reflect the conceptual breadth of social participation behaviours included. All items were recoded so that higher values indicate higher levels of participation or pro-social orientation. The Expressive profile reflects value-based orientations (e.g., equality, autonomy, and prosocial concern), while the Activating profile captures non-institutional political behaviours.

**Table S3.** Correlations between participation sub-indices

|  | Dutiful | Activating | Expressive | Cooperative |
| --- | --- | --- | --- | --- |
| Dutiful | 1.00 |  |  |  |
| Activating | 0.120 | 1.00 |  |  |
| Expressive | 0.056 | 0.114 | 1.00 |  |

| Cooperative | 0.166 | 0.243 | 0.209 | 1.00 |
| --- | --- | --- | --- | --- |

**Note:** Pearson correlation coefficients. All correlations are statistically significant at p < 0.01 (two-tailed). Differences in sample size (N) reflect item-specific missing data. The matrix is symmetric; only the lower triangle is displayed.
